# Novel players in organogenesis and flavonoid biosynthesis in cucumber glandular trichomes

**DOI:** 10.1093/plphys/kiad236

**Published:** 2023-04-26

**Authors:** Zhongxuan Feng, Lei Sun, Mingming Dong, Shanshan Fan, Kexin Shi, Yixin Qu, Liyan Zhu, Jinfeng Shi, Wujun Wang, Yihan Liu, Liyan Song, Yiqun Weng, Xingwang Liu, Huazhong Ren

**Affiliations:** Department of Vegetable Science, College of Horticulture, China Agricultural University, Beijing 100193, China; Department of Vegetable Science, College of Horticulture, China Agricultural University, Beijing 100193, China; Department of Vegetable Science, College of Horticulture, China Agricultural University, Beijing 100193, China; Department of Vegetable Science, College of Horticulture, China Agricultural University, Beijing 100193, China; Department of Vegetable Science, College of Horticulture, China Agricultural University, Beijing 100193, China; Department of Vegetable Science, College of Horticulture, China Agricultural University, Beijing 100193, China; Department of Vegetable Science, College of Horticulture, China Agricultural University, Beijing 100193, China; Department of Vegetable Science, College of Horticulture, China Agricultural University, Beijing 100193, China; Department of Vegetable Science, College of Horticulture, China Agricultural University, Beijing 100193, China; Department of Vegetable Science, College of Horticulture, China Agricultural University, Beijing 100193, China; Agricultural and Rural Bureau of Qingxian in Hebei Province, Qingxian 062650, China; USDA-ARS, Vegetable Crops Research Unit, Horticulture Department, University of Wisconsin, Madison, WI 53706, USA; Department of Vegetable Science, College of Horticulture, China Agricultural University, Beijing 100193, China; Sanya Institute of China Agricultural University, Sanya 572019, China; Engineering Research Center of Breeding and Propagation of Horticultural Crops, Ministry on Education, College of Horticulture, China Agricultural University, Beijing 100193, China; Department of Vegetable Science, College of Horticulture, China Agricultural University, Beijing 100193, China; Sanya Institute of China Agricultural University, Sanya 572019, China; Engineering Research Center of Breeding and Propagation of Horticultural Crops, Ministry on Education, College of Horticulture, China Agricultural University, Beijing 100193, China

## Abstract

Glandular trichomes (GTs) are outgrowths of plant epidermal cells that secrete and store specialized secondary metabolites that protect plants against biotic and abiotic stresses and have economic importance for human use. While extensive work has been done to understand the molecular mechanisms of trichome organogenesis in Arabidopsis (*Arabidopsis thaliana*), which forms unicellular, nonglandular trichomes (NGTs), little is known about the mechanisms of GT development or regulation of secondary metabolites in plants with multicellular GTs. Here, we identified and functionally characterized genes associated with GT organogenesis and secondary metabolism in GTs of cucumber (*Cucumis sativus*). We developed a method for effective separation and isolation of cucumber GTs and NGTs. Transcriptomic and metabolomic analyses showed that flavonoid accumulation in cucumber GTs is positively associated with increased expression of related biosynthesis genes. We identified 67 GT development–related genes, the functions of 7 of which were validated by virus-induced gene silencing. We further validated the role of cucumber ECERIFERUM1 (CsCER1) in GT organogenesis by overexpression and RNA interference transgenic approaches. We further show that the transcription factor TINY BRANCHED HAIR (CsTBH) serves as a central regulator of flavonoid biosynthesis in cucumber GTs. Work from this study provides insight into the development of secondary metabolite biosynthesis in multicellular GTs.

## Teaser

Glandular trichome-enriched genes function in both glandular trichome development and flavonoid metabolism, as revealed by transcriptomic and metabolic analyses.

## Introduction

Trichomes are hair-like outgrowths from epidermal cells that cover the aerial parts of plants. Based on their structure and function, trichomes are often classified as glandular trichomes (GTs) or nonglandular trichomes (NGTs) which may be unicellular or multicellular. For example, Arabidopsis (*Arabidopsis thaliana*) has unicellular NGTs, while the trichomes of tomato (*Solanum lycopersicum*) and cucumber (*Cucumis sativus*) or tobacco (*Nicotiana tabacum*) are multicellular GTs or multicellular NGTs, respectively. The organogenesis of various types of trichomes is controlled by different developmental programs with multiple evolutionary origins (Serna and Martin 2006). Trichomes may play important roles in protecting plants from environmental stresses such as heat, low temperature, high UV radiation, and insect herbivory (Wagner 1991; Werker 2000; Karaourniotis et al. 2020; Schuurink and Tissier 2020). The GTs have received much attention in research because they are the site of biosynthesis and storage of specialized secondary metabolites such as flavonoids, alkaloids, terpenoids, phenylpropanoids, methyl ketones, and acyl sugars; many of these compounds are of great importance for human use as pharmaceuticals, flavor and fragrance ingredients, or pesticides (reviewed in Fahn 2000; Huchelmann et al. 2017; Schuurink and Tissier 2020; Feng et al. 2021). For example, the GTs of cannabis (*Cannabis sativa*) flowers produce massive quantities of cannabinoids and terpenoids which have valuable pharmacological properties and constitute a multibillion-dollar industry worldwide (Livingston et al. 2020). The sweet wormwood (*Artemisia annua*) GTs produce artemisinin, which is used for treating malaria (Hassani et al. 2020). GTs are present in ∼30% of vascular plant species which originate from epidermal cells that are easy to acquire and observe making them an ideal model for studying cell differentiation (Yang and Ye 2013; Schuurink and Tissier 2020).

The *Arabidopsis* unicellular NGT has long been a model system to study the molecular genetic mechanisms of trichome organogenesis, which involves a transcriptional network consisting of several groups of transcription factors (TFs) such as R2R3 MYBs, the basic helix–loop–helix (bHLH), and the WD40 repeat (WDR) proteins (reviewed by Wang et al. 2019). Investigations on GT organogenesis and secondary metabolisms in GT in nonmodel species are limited, but substantial progress has been made recently in several economically important crop plants like tomato, cucumber, and sweet wormwood (reviewed in Chalvin et al. 2020; Feng et al. 2021). Several master TFs that play important regulatory roles in GT organogenesis and/or secondary metabolisms have been identified, which are members of the APETALA2/ethylene responsive factor (AP2/ERF) family (Wang et al. 2023), the bHLH family (Xu et al. 2018; Chen et al. 2020), the MYB family (Yang et al. 2018; Gong et al. 2021; Yuan et al. 2021), the homeodomain-leucine zipper (HD-ZIP) family (Cui et al. 2016; Xie et al. 2020; Hua et al. 2021; Zhang et al. 2021), or the zinc finger protein (ZFP) family (Chang et al. 2018; Chun et al. 2021; Hua et al. 2022). There are also studies to investigate genetic control and regulation of biosynthesis of secondary metabolites in GTs. Some examples include terpenoid synthesis in type Ⅵ GTs of tomato (Ewas et al. 2016; Gong et al. 2021; Yang et al. 2021), artemisinin synthesis in GTs of sweet wormwood (*A. annua*) (Shen et al. 2016; Chen et al. 2017; Hassani et al. 2020), cannabinoid synthesis in GTs of cannabis (Livingston et al. 2020), and biosynthesis of metabolites in some medicinal plants (Zhang et al. 2015; Liu, Wu, et al. 2016; Liu et al. 2018).

Cucumber is an economically important vegetable crop. Some attributes of trichomes, especially fruit trichomes (spines) such as their presence or absence, density, and size, constitute important criteria in classification of market groups (Weng 2021). Eight types of fruit trichomes have been recognized with the majority belonging to Type I GTs and Type II NGTs; Type II NGTs are large and are visible spines on cucumber fruits, whereas the Type I peltate GTs are small with a short stalk comprising 3 or 4 cells and a 4-celled head (Liu et al. 2016; Xue et al. 2019). Several studies in cucumber have identified 2 hub TFs with pivotal roles in trichome development including *tiny branched hair* (*CsTBH*) (syn., *microtrichome*/*CsMict*, *glabrous1*/*CsGL1*) (Chen et al. 2014; Li et al. 2015; Zhao et al. 2015; Pan et al. 2021; Zhang et al. 2021) and *CsGL3* (*glabrous3*) (syn. *trichome-less*/*CsTril*) (Pan et al. 2015; Cui et al. 2016; Wang et al. 2016). *CsGL3* encodes a HD-Zip IV TF that is important in trichome initiation (Pan et al. 2015; Cui et al. 2016; Wang et al. 2016). *CsTBH* encodes a HD-Zip I TF. The *tbh* mutant has no noticeable spines but contains papillae on the leaf epidermis suggesting that it may also function in the early stage of trichome development (Pan et al. 2015; Cui et al. 2016; Wang et al. 2016). These cucumber trichome development–related TFs seem to play roles in development of both GTs and NGTs. No GT development–specific genes or genes regulating secondary metabolism inside GTs have been identified in cucumber. In our recent study, the development of cucumber cotyledon NGTs and GTs was classified into 5 stages, and a set of trichome development–related genes was identified from transcriptomic analysis (Dong et al. 2022). However, whether these genes function in the morphogenesis of GTs, NGTs, or both is unknown.

Both GTs and NGTs are present on cucumber aerial organs. Investigation of GT-specific development and metabolic pathways requires an efficient method to separate and isolate GTs and NGTs, which was not available in cucumber. In *A. annua*, laser capture microdissection and pressure catapulting were used to isolate GTs (Olofsson et al. 2012). Bergau et al. described a method for the separation and isolation of intact young and mature type VI trichomes from the wild tomato (*Solanum habrochaites*) using autofluorescence-based cell sorting (Bergau et al. 2016). Both methods seem technically demanding and time-consuming. In the present study, we report a modified method to separate and isolate cucumber GTs and NGTs, which were used to investigate GT and NGT transcriptomes through RNA-sequencing (RNA-Seq). We also conducted metabolomic analysis in GTs through ultrahigh-performance liquid chromatography–tandem mass spectrometry (UHPLC–MS). These analyses revealed metabolites, metabolic pathways, and associated regulatory genes enriched in cucumber GTs. We identified a set of GT development–related genes (GTRGs), and their functions were validated by virus-induced gene silencing (VIGS). The roles of the cucumber *ECERIFERUM1* (*CsCER1*) gene in GT organogenesis were further validated with overexpression (OE) and RNAi transgenic approaches. We further show that *CsTBH* is involved not only in trichome development (Zhang et al. 2021) but also in the regulation of flavonoid biosynthesis in GTs.

## Results

### Development of an efficient protocol for separation and isolation of GTs and NGTs in cucumber

**Figure 1.**
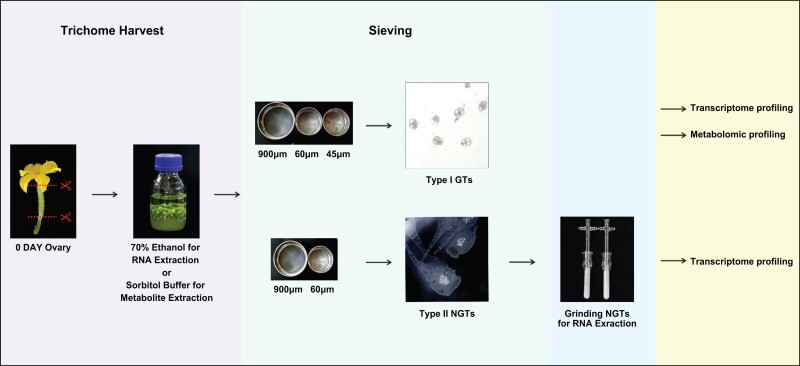
Workflow for the separation and isolation of cucumber GTs and NGTs.

We developed a protocol to separate and isolate GTs and NGTs in cucumber by modifying a previously reported method in tomato (Bergau et al. 2016). In our early study, we found that the majority of trichomes (spines) in different cucumber organs (fruits, leaves, petioles, stems, etc.) belong to Type Ⅰ GTs and Type II NGTs, and the mean width of GTs and NGTs is around 30 and 700 *µ*m, respectively (Xue et al. 2019). In cucumber, the trichomes (spines) on developing ovaries near anthesis are very dense and large, which is particularly true in modern Chinese Long varieties that are characterized with high-density fruit spines. Thus, to maximize yield, we collected trichome samples from the ovaries on the day of anthesis (0 d after flowering [DAF]). A flow chart is shown in Fig. 1. GTs and NGTs could be well separated by passing the trichome samples sequentially through 900 *µ*m + 60 *µ*m and 900 *µ*m + 60 *µ*m + 45 *µ*m steel sieves, respectively. The isolated NGTs were subjected to further grinding for RNA-Seq. The identities of isolated GTs and NGTs were verified through microscopic examinations (Fig. 1). We also evaluated the quantity of the GT and NGT samples. We counted GTs in the sample using a hemocytometer and estimated that the concentration of the isolated GTs was ∼10^6^/mL. The concentration of total RNAs prepared from GT and NGT samples reached 900 and 60 ng/*µ*L, respectively. These data suggest that the yield and quality of GTs and NGTs obtained with our method are adequate for downstream analyses.

### Transcriptomic profiling identifies DEGs in the flavonoid biosynthesis pathway enriched in cucumber GTs

**Figure 2.**
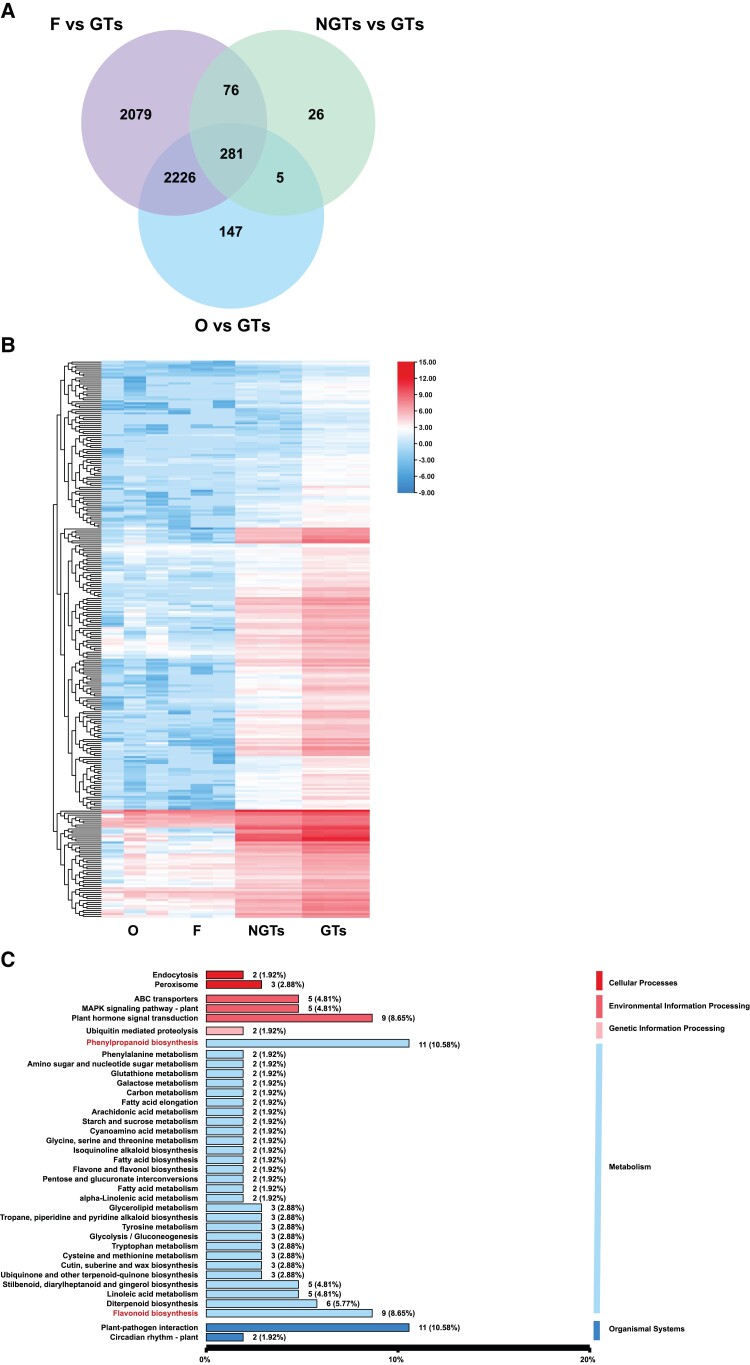
Upregulated genes enriched in the GT transcriptome based on RNA-Seq. **A)** Venn diagram of upregulated genes among 4 transcriptomes. **B)** Heat map of expression levels of GT-enriched and upregulated genes as compared with from other organs. **C)** KEGG classification pathways inferred from GT-enriched genes. O, ovaries without trichomes; F, fruit flesh; GTs, glandular trichomes; NGTs, non-glandular trichomes.

We performed RNA-Seq of the isolated GTs and NGTs. To identify differentially expressed genes (DEGs) in GTs, the transcriptomes of trichomes of the glabrous ovaries (O) and its flesh (F) were also sequenced. RNA-Seq of 12 samples (3 reps per sample) generated ∼520 million reads, of which 90% could be uniquely mapped on the 9930 v2.0 draft genome (Supplemental Table S1). From 3 pair-wise comparisons (NGTs vs. GTs, F vs. GTs, and O vs. GTs), with |log_2_(fold change)| ≥ 1 and false discovery rate (FDR) < 0.01 as the cutoffs, we identified 423 genes that were differentially expressed in GTs as compared with 3 other samples, 281 of which were upregulated (Fig. 2, A and B, and Supplemental Table S2). These GT-enriched genes may play important roles in GT development and metabolism (e.g. Shi et al. 2018) which were thus our focus in subsequent analyses. Kyoto Encyclopedia of Genes and Genomes (KEGG) enrichment analysis of the 281 DEGs indicated that they were involved in 63 KEGG pathways (Fig. 2C and Supplemental Table S3). Among them, the most enriched pathways were phenylpropanoid/flavonoid biosynthesis (20%), plant–pathogen interaction (11%), and phytohormone signal induction (9%). Phenylpropanoid biosynthesis is the precursor process for flavonoid biosynthesis. Flavonoids are widely synthesized in plant GTs that may contribute to resistances against insects or pathogens and abiotic stress tolerance. Thus, cucumber GTs accumulating flavonoids may play a role in biotic and abiotic stress tolerances.

Among the 281 DEGs enriched in GTs, 22 were TF genes from 14 families (Supplemental Table S4), most of which have been reported to be involved in the development of GTs and metabolite biosynthesis in GTs. Among the 22 TFs, 5 belong to the AP2/ERF family, which is 1 of the largest plant TF families and consists of TFs characterized by conserved AP2/ERF DNA binding domains of 57 to 66 amino acids in size (Okamuro et al. 1997). Some AP2/ERF TFs regulate GT morphogenesis and control the expression of multiple genes involved in metabolite biosynthesis in GTs (Lu et al. 2013; Tan et al. 2015; Shen et al. 2016).

### Accumulation of flavonoids in cucumber GTs is positively associated with increased expressions of relevant biosynthesis genes

**Figure 3.**
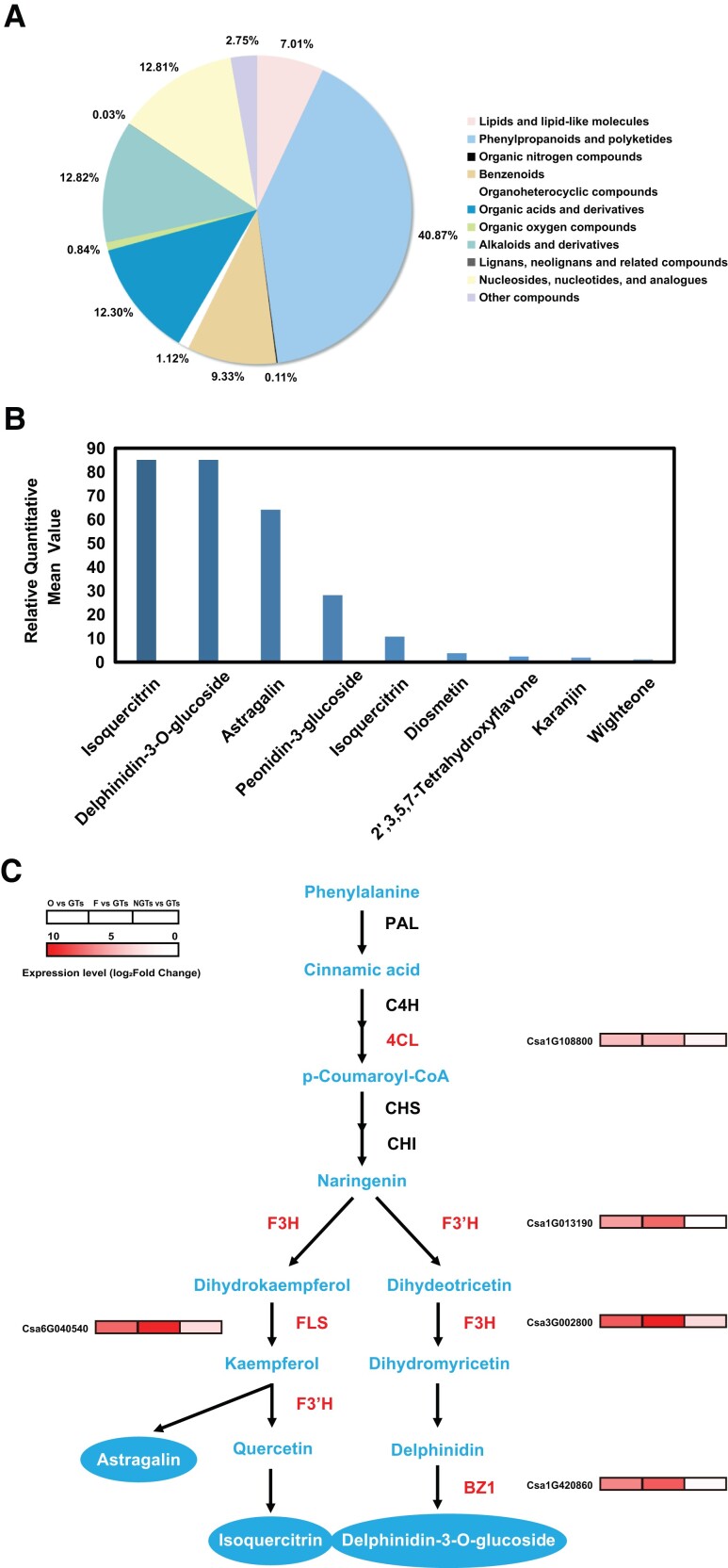
Flavonoids and biosynthetic genes in the flavonoid biosynthesis pathway enriched in GTs. **A)** Distribution of 11 groups of 711 metabolites identified from cucumber GTs. **B)** Flavonoids with high relative content in GTs. **C)** DEGs (*4CL*, *F3H*, *F3'H*, *FLS*, *BZ1*) for enzymes in the flavonoid biosynthesis pathway. The heat maps by each gene indicated fold changes of the gene from RNA-Seq data. The 3 rectangles in each heat map represent (left to right) the value of log_2_(fold change) in the comparisons of O versus GTs, F versus GTs, and NGTs versus GTs, respectively. O, ovaries without trichomes; F, fruit flesh; GTs, glandular trichomes; NGTs, non-glandular trichomes.

We further conducted metabolomic analysis in the isolated GTs with UHPLC–MS. We obtained 744 metabolites (Supplemental Table S5) which could be roughly classified into 11 groups based on the Human Metabolome Database (HMDB) super class classification (Fig. 3A). Among them, the top 3 largest groups of metabolites belonged to lipids and lipid-like molecules (193 or 27.1%), phenylpropanoids and polyketones (158 or 22.2%), and benzenoids (83 or 11.3%) (Fig. 3A and Supplemental Table S5). Although there are various kinds of lipids and lipid-like molecules in GTs, phenylpropanoids and polyketones had the highest percentage of total relative content, accounting for 40.87% of all compounds (Fig. 3A). These data were consistent with results from transcriptomic analysis that the flavonoid biosynthesis pathway was highly active in cucumber GTs.

Further subclassification indicated that 108 of the 158 (68.3%) phenylpropanoid and polyketide metabolites were flavonoids. Among these flavonoids, isoquercitrin, delphinidin-3-*O*-glucoside, and astragalin exhibited the highest contents (Fig. 3B). Major steps of the flavonoid biosynthetic pathway for biosynthesis of these flavonoids are illustrated in Fig. 3C. Key enzymes for biosynthesis of these flavonoids include phenylalanine ammonia-lyase (PAL), cinnamate 4-hydroxylase (C4H), 4-coumaroyl CoA ligase (4CL), chalcone synthase (CHS), chalcone isomerase (CHI), flavanone 3-hydroxylase (F3H), flavonoid 3′-hydroxylase (F3′H), flavonol synthase (FLS), and anthocyanidin 3-*O*-glucosyltransferase (BZ1). We found that the expression of genes for 4CL, F3H, F3′H, FLS, and BZ1 (highlighted in red in Fig. 3C) was substantially increased in GTs as compared with their expression in ovaries without trichomes (O), fruit flesh (F), and NGTs, which could be seen from the log_2_(fold change) value of each gene based on transcriptomic data (Fig. 3B). The expression patterns of these genes were also validated by reverse transcription quantitative PCR (RT-qPCR) (Supplemental Fig. S1). These data demonstrate a positive association between the high expression of flavonoid synthesis–related genes and the accumulation of flavonoids in GTs.

### CsTBH regulates the expression of genes in the flavonoid biosynthesis pathway in cucumber GTs

**Figure 4.**
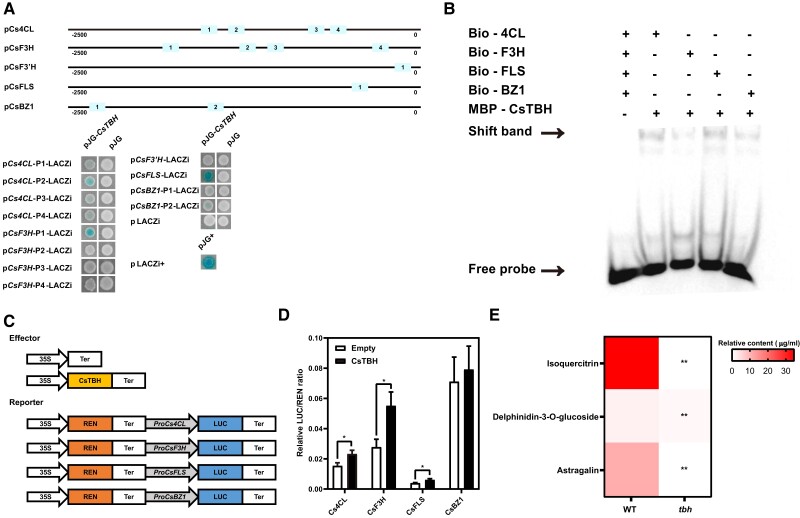
CsTBH regulates flavonoid biosynthesis in GTs through its direct binding to the promoters of biosynthetic genes. **A)** Y1H assays suggest that CsTBH binds to the promoters of 4 flavonoid biosynthesis genes. Boxes indicate the binding motifs CAATTAT and CAATAAT. **B)** EMSA experiments further support that CsTBH binds to the promoters of the 4 genes. **C** to **D)** Schematic diagram of the reporters and effectors used in the LUC assay showing CsTBH activates the expression of 4 flavonoid biosynthesis genes. The LUC/REN ratio represents the LUC activity relative to the internal control. **E)** Heat map showing the relative contents of 3 flavonoids with high relative content in GTs in WT and *tbh* lines by HPLC. Error bars represent Sd from 3 biological reps. * indicates *P* < 0.05. ** indicates *P* < 0.01.

One upregulated gene in GTs (Supplemental Table S4) is *CsTBH* (*Csa3G748220*), a HD-ZIP–type TF gene and a key regulator of cucumber trichome development (Zhang et al. 2021). *CsTBH* functions during early trichome development; loss of function mutation of this gene results in a tiny branched hair phenotype that cannot distinguish if it is GT or NGT (Zhang et al. 2021; Dong et al. 2022). The cucumber *tbh* mutant also shows a substantial reduction in flavonoid accumulation as compared with its wild type (Pan et al. 2021). From data in this study, we speculate that *CsTBH* may regulate flavonoid biosynthesis in cucumber GTs by regulating the expression of relevant genes in this pathway. To confirm this, we performed yeast 1-hybrid (Y1H) assays to examine the binding of CsTBH to the promoters of selected genes. We first analyzed potential signature binding motifs (CAATTAT and CAATAAT) of CsTBH in the promoters of *Cs4CL*, *CsFLS*, *CsF3H*, *CsF3′H*, and *CsBZ1* that were highly expressed in GTs. We found each of the 5 genes had 1 to 4 such binding motifs (Fig. 4A). Y1H assays revealed that CsTBH was able to bind the motifs in the promoter of all genes except *CsF3′H* (Fig. 4A). These Y1H results were confirmed with electrophoretic mobility shift assay (EMSA) experiments (Figs. 4B and S2) although the binding ability between CsTBH and the promoter of *CsBZ1* was somewhat weaker. We further conducted dual-luciferase (LUC) transactivation assays in *Nicotiana benthamiana* leaves and found that the intensity of LUC signals was significantly increased when 35S:*CsTBH* was cotransformed with p*Cs4CL*:LUC, p*CsF3H*:LUC, and pCs*FLS*:LUC (Fig. 4, C and D).

We measured the contents of 3 flavonoids, isoquercitrin, delphinidin-3-*O*-glucoside, and astragalin, in GTs of the *tbh* mutant and its wild type using HPLC (Fig. 3B), and we found that all 3 flavonoids were decreased significantly in *tbh* compared with in the WT (Fig. 4E). Taken together, these data support an important role of *CsTBH* in regulating both GT development and flavonoid biosynthesis in GTs.

### Comparative transcriptomic analysis and VIGS assays identify critical genes involved in GT morphogenesis

Genes expressed specifically in GTs are likely to regulate the development and secondary metabolisms of GTs (Shen et al. 2018). However, most of the 281 GT-enriched DEGs (Supplemental Table S2) were involved in various metabolite biosynthesis processes. In our early study (Dong et al. 2022), we conducted transcriptome profiling on trichomes of cotyledons at different development stages and identified a number of genes related to cucumber multicellular trichome development. However, whether they play any role in GTs or NGTs or both is not clear. Our GT- and NGT-specific transcriptomic data from this study may clarify this. To identify GTRGs, we conducted a joint analysis of 281 GT-enriched genes from the present study with 711 multicellular trichome–related genes from our early study (Dong et al. 2022; Supplemental Table S3). This analysis resulted in 67 GTRGs (Supplemental Table S6). Among them were 4 TF genes: *CsMIF3* (*Csa1G009710*, a homolog of Arabidopsis *MINI ZINC FINGER 3* and a member of the ZF-HD TF family), *CsWIN1-like* (*Csa2G006270*, a homolog of Arabidopsis *WAX INDUCER 1/SHINE1* or *WIN1/SHN1*), *CsESR2-like* (*Csa2G34909*0, a homolog of Arabidopsis *ENHANCER OF SHOOT REGENERATION 1*/*ESR1*) of the AP2/ERF TF family, and *CsTBH* of the HD-ZIP TF family.

We evaluated the possible roles of 3 of the 4 TFs (all except *CsTBH*) in GT development through tobacco ringspot virus (TRSV)–based VIGS. We also included 5 additional genes in our transient VIGS assays which seem to play roles in GT development including *CsCER1* (*Csa6G079750*, Arabidopsis homolog of *ECERIFERUM 1/CER1*), *CsFOCL1* (*Csa2G264000*, Arabidopsis homolog of *FUSED OUTER CUTICULAR LEDGE1/FOC1*), *CsGIR*2 (*Csa6G425780*, Arabidopsis homolog of *GIR2*), *CsMBOAT1* (*Csa7G091730*, Arabidopsis homolog of *membrane bound O-acyl transferase1/MBOAT1*), and *CsABCG40* (*Csa7G433950*, Arabidopsis homolog of *ATP-BINDING CASSETTE G40/ABCG40*) (see below for discussion of functions of these genes in GT development). For each gene, VIGS of the cucumber *phytoene desaturase* (*CsPDS*, *Csa4G011080*) gene was used as the positive control (TRSV::*CsPDS*), which resulted in a photo-bleaching phenotype (Supplemental Fig. S3). Empty TRSV-infected wild-type XTMC plants (TRSV::00) were used as the negative control.

**Figure 5.**
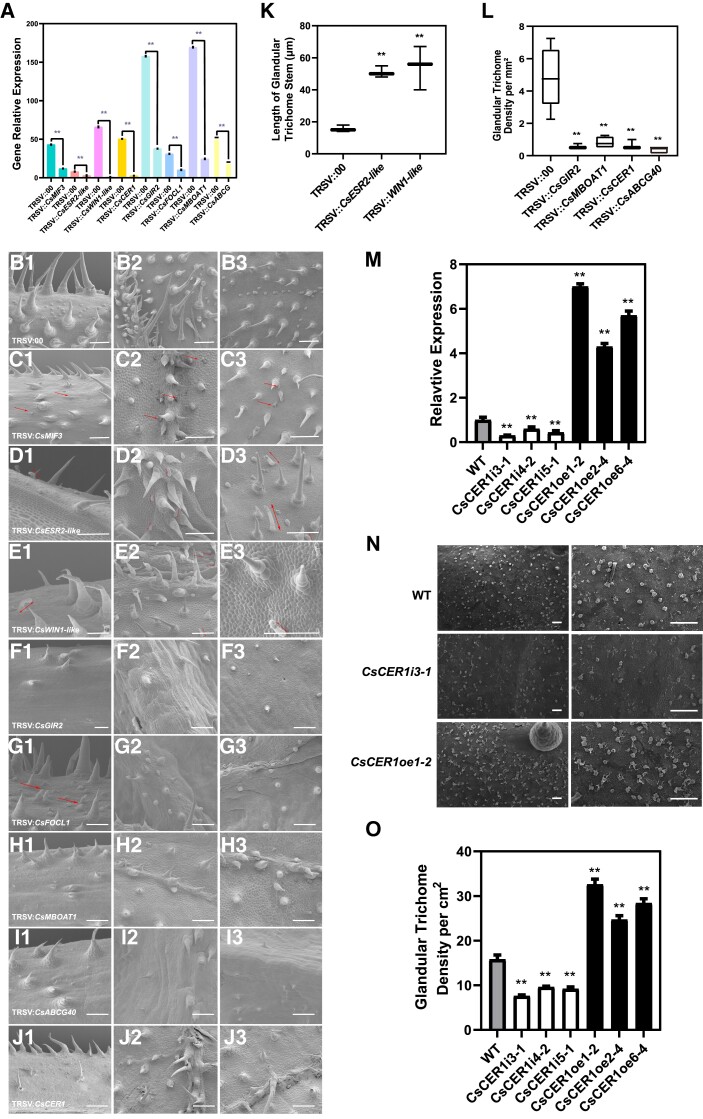
Functional characterization of selected GTRGs in cucumber. **A)** Gene expression levels in VIGS plants. *CsTUA* (α*-tubulin*) served as the internal control. **B** to **J)** Trichome phenotypes of VIGS plants: TRSV::00 (B1 to B3), TRSV::*CsMIF3* (C1 to C3), TRSV::*CsESR2-like* (D1 to D3), TRSV::*CsWIN1-like* (E1 to E3), TRSV::*CsGIR2* (F1 to F3), TRSV::*CsFOCL1* (G1 to G3), TRSV::*CsMBOAT1* (H1 to H3), TRSV::*CsABCG40* (I1 to I3) ,and *TRSV::CsCER1* (J1 to J3). For the panel of each gene, the first image (left) is taken from the petiole of the first true leaf, and the other 2 are both from the first true leaf. The arrows indicate the abnormal GTs. **K)** The stem length of GTs in different VIGS plants. The plot box represents the median (center line) and upper and lower quartiles (box limits), and whiskers extend to the minimum and maximum values within 1.5 × interquartile range. **L)** The density of GTs (per mm^2^) in different silenced plants. The plot box represents the median (center line) and upper and lower quartiles (box limits), and whiskers extend to the minimum and maximum values within 1.5 × interquartile range. **M)** RT-qPCR analysis of *CsCER1* expression in *CsCER1*-RNAi and *CsCER1*-OE lines. **N)** GT phenotypes on 8 DAF fruits in *CsCER1*-RNAi and *CsCER1*-OE lines. **O)** GT density (per cm^2^) in *CsCER1*-RNAi and *CsCER1*-OE lines. Bars represent 250 *µ*m. Error bars represent mean ± Sd (*n* = 3 biological reps). ** indicates *P* < 0.01.

For each of the 8 genes, RT-qPCR revealed their significantly lower expression in the VIGS plant than in the negative control (Fig. 5A) suggesting that these genes were effectively silenced. In TRSV::00 plants, the GTs were dense on the leaf veins and petioles (Fig. 5B), so we sampled the petioles and leaf blade with main vein for phenotypic characterization of the VIGS plants. Most GTs on TRSV::*CsMIF3* plants showed a shrinking secretory head (Fig. 5C) suggesting that silencing of *CsMIF3* leads to GT dysplasia. VIGS of the 2 AP2/ERF TF genes showed a similar phenotype. The GTs on plants infected with TRSV::*CsESR2-like* or TRSV::*CsWIN1-like* showed elongated stems (Fig. 5, D, E, and K). Plants infected with TRSV::*CsGIR2*, TRSV::*CsCER1*, TRSV::*CsMBOAT1*, and TRSV::*ABCG40* all showed a significant decrease in GT density (Fig. 5, F, H to J, and L). The phenotype of TRSV::*CsFOCL1* plants was intriguing. We found that the morphogenesis of many NGTs on TRSV::*CsFOCL1* plants stopped at the raised stage, whereas the development of GTs was not affected (Fig. 5G).

VIGS represents only the transient silencing of a gene, which may not reveal its specific functions. For example, a knockout line of *CsTBH* affects the development of both GTs and NGTs, but in this VIGS system, its effect on GTs was very weak (Zhang et al. 2021; Dong et al. 2022). Therefore, we further developed transgenic lines OE and RNAi of the *CsCER1* gene (Fig. 5M). At 8 DAF, we examined GT density on the fruit peels from OE and RNAi lines and found that OE of *CsCER1* significantly increased the density of GTs while the knockdown of *CsCER1* significantly decreased the density of GTs (Fig. 5, N and O).

## Discussion

### Cucumber GTs actively synthesize flavonoids

Our metabolomic and transcriptomic analyses confirmed that cucumber GTs were able to synthesize flavonoids. So far, 57 flavonoids have been identified in plant GTs including artemetin, naringenin, apigenin, and kaempferol (Liu et al. 2019). In the present study, we found that the 3 substantially accumulated flavonoids in cucumber GTs were astragalin, isoquercitrin, and delphinidin-3-*O*-glucoside (Fig. 3B).

Astragalin, isoquercitrin, and delphinidin-3-*O*-glucoside are antioxidants which may be involved in the production and removal of reactive oxygen species (ROS) and contribute to biotic or abiotic stress tolerances (Emiliani et al. 2013; Maloney et al. 2014; Riaz et al. 2018). Thus, in cucumber, we speculate that GTs may contribute to chemical resistance to pathogen infection or insect herbivory by storing and secreting these antioxidants.

### Involvement of *CsTBH* in regulation of both GT development and flavonoid biosynthesis in cucumber GTs

*CsTBH1* is a member of the HD-Zip I TF family and a key regulator in trichome development and sex expression in cucumber (Li et al. 2015; Zhao et al. 2015; Zhang et al. 2021). In a previous study, the cucumber *tbh* mutant was reported to have a decreased flavonoid accumulation as compared with the WT suggesting *CsTBH* may regulate flavonoid biosynthesis (Pan et al. 2021). In this study, we found that *CsTBH* was highly expressed in GTs which had elevated flavonoid accumulation. Y1H, EMSA, and dual-LUC assays revealed that CsTBH can bind to the promoters of several important genes in the flavonoid biosynthesis pathway to regulate their expression (Fig. 4, A to D). Besides, the 3 most abundant flavonoids in cucumber GTs including astragalin, isoquercitrin, and delphinidin-3-*O*-glucoside were significantly decreased in the *tbh* mutant compared with WT (Fig. 4E). These data support a role of *CsTBH* in modulating both GT organogenesis and flavonoid biosynthesis in GTs in cucumber.

### Novel players in cucumber GT development

From transcriptome analysis among multiple transcriptomes, we identified 67 GTRGs including 4 TF genes: *CsTBH*, *CsWIN1-like*, *CsESR2-like*, and *CsMIF3* (Supplemental Table S6). As discussed above, *CsTBH* seemed to play a key role in both GT development and secondary metabolism in GTs. *CsWIN1-like* is a homolog of Arabidopsis *WIN1*, a member of the ERF (ethylene response factor) subfamily B-6 of ERF/AP2 TF family. In *Arabidopsis*, WIN1 interacts with MIXTA-like TFs to regulate wax biosynthesis and cuticle development (Oshima et al. 2013). In *A. annua*, the WIN1 homolog regulates GT development (Tan et al. 2015). The cucumber *CsWIN1-like* gene (syn. *CsSHN1*) seems to be involved in epidermal wax biosynthesis (Li et al. 2018). Based on data from the present study, *CsWIN1* may play a role in GT development (Fig. 5E). Recently, the *CsESR2-like* homolog *SlLFS* was reported to promote the differentiation of peltate trichomes in tomato (Wu et al. 2023). In this study, the peltate GTs of *CsESR2-like* VIGS plant exhibited an elongated stem that appeared to shift in the direction of capitate GTs, suggesting that this class of AP2/ERF TFs may be conserved in tomato and cucumber for GT development (Fig. 5D). In *Arabidopsis*, *AtMIF3* functions in meristem formation (Hu et al. 2011). Its homolog in tomato seems to be involved in the termination of floral meristem (Bollier et al. 2018). Our VIGS work suggests possible involvement of *CsMIF3* in cucumber GT development. However, additional work is needed to confirm this.

In addition to the 4 TFs, we also performed VIGS assays for 5 more genes that may be involved in GT development including *CsGIR2*, *CsFOC1*, *CsCER1*, *CsMBOAT1*, and *CsABCG40* (Fig. 5). In *Arabidopsis*, AtGIR2 functions as an adaptor protein interacting with trichome formation–related AtGL2 to regulate root hair formation (Wu and Citovsky 2017). The *Arabidopsis AtFOCL1* encodes a protein secreted by guard cells and functions in epidermal development (Hunt et al. 2017). *CsCER1*, *CsMBOAT1*, and *CsABCG40* are all lipid metabolism–related genes. Previous studies suggest that lipids may act as signals in the regulation of plant trichome development because biosynthesis of cutin and wax is inextricably linked to the development of trichomes (Berhin et al. 2022). In the present work, many lipid transport and metabolism-related genes were highly expressed in GTs and many lipids and lipid-like compounds were also enriched in GTs (Fig. 2C and Supplemental Table S5).

In Arabidopsis, *cer1* and *cer3* double mutant shows deformation of trichomes (Kurata et al. 2003). In cucumber, *CsCER1* has been shown to influence cuticle properties and VLC (very long chain) alkane biosynthesis (Wang et al. 2015). Here, transgenic cucumber plants OE *CsCER1* showed increased GT density, while RNAi plants silencing *CsCER1* resulted in decrease of GT density, which was consistent to the phenotype observed in VIGS plants of this gene (Fig. 5, M to O). These data strongly support a role of *CsCER1* in cucumber GT development. This is not surprising because the subtle interplay between trichome development and cuticle formation in plants suggests a substantial role for epidermal morphogenesis–related genes in GT development (Berhin et al. 2022).

Although *CsFOCL1* is highly expressed in GTs, the results from VIGS assay suggest that it appears to play a role in NGT development. This is possible because genes enriched in GTs may also regulate NGT development such as *CsTBH*. *CsMBOAT1* is a long-chain–alcohol *O*-fatty-acyltransferase that may be involved in epidermal wax biosynthesis (Li-Beisson et al. 2013). Adenosine triphosphate (ATP)–binding cassette transporters (ABCG) are important in cutin biosynthesis (Schuurink and Tissier 2020). Thus, we isolated *CsABCG40*, the only ABC transporter from 67 GRTGs, for further function validation. While our data established a function link of these genes with GT development, the specific functions in this process need further work.

Finally, we used VIGS to examine the functions of selected GTRGs. The VIGS technology has relatively low efficiency especially in cucumber fruits. This was the reason we examined the morphological characteristics of trichomes in VIGS plants using leaf/petiole samples at the seedling stage, while the transcriptomes and metabolomes were analyzed using ovaries. The distribution and morphology of cucumber GTs and NGTs do not seem to have substantial differences in different organs (Xue et al. 2019). The difference of trichome development between different organs has not been thoroughly studied, but several previous studies on *CsGL3*, *CsTBH*, and *CsCER1* did not find organ-specific functional variation of these genes in cucumber (Cui et al. 2016; Zhang et al. 2021). Nevertheless, like *SlHair2* in tomato (Chun et al. 2021), the tissue-specific glandular development regulatory gene in cucumber merits further investigations.

## Materials and methods

### Plant materials and sample preparation

The main subjects of the present study included the North China type (Chinese Long) cucumber (*C. sativus* L.) inbred line Xintaimici (XTMC), the *tbh* mutant, and its wild type (Zhang et al. 2021). Seeds of these materials were from the senior authors' lab. All plants were grown in a greenhouse of China Agricultural University in Beijing. Pest control and water management followed standard practices.

Trichomes were harvested by a bead-beating method modified from a previous study (Bergau et al. 2016). On the day of anthesis, 3 sets of 25 to 30 each developing ovaries from XTMC plants were harvested, and the middle section (∼1.0 cm in length) were cut and collected in 250 mL glass bottles containing either ice-cold sorbitol buffer [200 mM sorbitol, 50 mM Tris–HCl, 20 mM sucrose, 10 mM KCl, 5 mM MgCl_2_, 5 mM succinic acid, 1 mM EGTA, 0.5 mM K_2_HPO_4_, and 0.015% (*v*/*v*) Triton X-100] for further extraction of metabolites or ice-cold 70% (*v*/*v*) ethanol for RNA-Seq. To isolate the GTs, 30 g of 1 mm glass beads was added to each bottle and then handshake for 15 min, followed by passing the mixture sequentially through 900, 60, and 45 *µ*m steel sieves. The filtrate was transferred to a 50 mL centrifuge tube. The supernatant was removed after centrifugation at 5,000 × *g* for 5 min, and the resulting precipitate consisted of GTs, which were either immediately used for RNA extraction or were flash frozen with liquid nitrogen and stored at −80 °C for late use.

The NGTs were collected from the top of the 60 *µ*m sieve and were concentrated by centrifugation for 5 min at 5,000 × *g*, with subsequent grinding by a tissue grinder. The supernatant was removed after centrifugation at 5,000 × *g* for 5 min, and the resulting precipitate consisted of the NGTs fraction for RNA extraction. The glabrous ovaries were directly collected from the top of the 45 *µ*m sieve. And part of the glabrous ovaries was peeled to harvest flesh.

### Scanning electron microscopy

Samples were fixed with 2.5% (*v*/*v*) glutaraldehyde at 4 °C for ∼24 h, washed with PBS (pH 7.2) 3 times, and postfixed in 1% (*v*/*v*) OsO_4_. The samples were then dehydrated through an ethanol series [30, 50, 70, 80, 90, and 100% (*v*/*v*), 3 times], critical point dried using a desiccator (HCP-2; Hitachi), and coated with gold palladium (EIKO IB-3). Images were taken with a Hitachi S-4700 scanning electron microscope (SEM) using a 2 kV accelerating voltage.

### Measurement and counting of GTs

Measurement of the stem length of GTs and count of GT numbers was conducted through analysis of SEM images by ImageJ software (Schroeder et al. 2021). For each sample, data were collected from 3 biological replications and 3 technical replicates for quantity statistics.

### VIGS assay and phenotypic observation

A modified TRSV-based VIGS (Fang et al. 2021) assay was performed to analyze the potential roles of selected GT-related genes (GTRGs) in cucumber. In brief, the coding sequence (CDS) (300 to 500 bp) of the target gene was inserted into the *Sna* BI restriction site of pTRSV2 and then transformed into *Agrobacterium tumefaciens* (strain GV3101). When the primary roots of germinating cucumber seeds reached ∼1 cm, the seeds were infected with mixed pTRSV1 and pTRSV2 (containing different target fragments) by vacuum infiltration under −900 kPa for 5 min. The seeds were then put on half MS solid media containing 100 *µ*M acetosyringone until the agrobacterium was visible around the seeds. The seedlings were transplanted into half Hoagland solution for 15 d.

There were 12 VIGS positive plants for each gene. The leaf blade with main vein and petiole of the first true leaf was collected from each seedling. We sampled all VIGS plants to observe the phenotype under an SEM and conduct RT-qPCR.

### Cucumber transformation for OE and RNAi of *CsCER1*

To generate the OE construct, the full-length CDS of *CsCER1* was PCR amplified and inserted into the *Bam* HI and *Sma* I restriction sites of the pBI121 vector under the control of the CaMV 35S promoter. The construct was then transformed into the XTMC using a cotyledon transformation method (Wang et al. 2014).

The *CsCER1* CDS was used to generate the CsCER1-RNAi construct using gene-specific primers with *Asc* I (5′ end) and *Swa* I (3′ end) as well as *Spe* I (5′ end) and *Bam* HI (3′ end) restriction sites. Both fragments were inserted in reverse orientation into the pFGC1008 vector, which was then transformed into XTMC. Each recombinant construct was introduced into the *Agrobacterium* strain GV3101 before cucumber transformation. The primers are listed in Supplemental Table S7.

### RT-qPCR and RNA-Seq

Total RNA was isolated using an RNA extraction kit (Huayueyang, Beijing, China) and then reverse transcribed using a PrimeScript Reagent Kit with gDNA Eraser (TaKaRa, Shiga, Japan). RT-qPCR was conducted in 96-well plates with an ABI 7500 Real-Time PCR System (Applied Biosystems, Waltham, MA, USA) using SYBR Premix Ex Taq (TaKaRa, Shiga, Japan). For each sample, there were 3 biological and 3 technical replicates. The cucumber α*-tubulin* gene *(Csa4G000580*) was used as the reference. The gene-specific primers used for RT-qPCR are provided in Supplemental Table S7.

For RNA-Seq, 1 *µ*g total RNA from each sample was used as the input material. Sequencing libraries were generated using NEB Next Ultra RNA Library Prep Kit (NEB, USA). High-throughput sequencing of the cDNA libraries was performed through commercial service on an Illumina platform. After filtering, high-quality paired-end sequencing reads were mapped to the cucumber 9930 v2.0 reference genome (http://cucurbitgenomics.org/ftp/genome/cucumber/Chinese_long/v2/) using HISAT (Kim et al. 2015). Normalized gene expression level was calculated as fragments per kilobase (FPKM) of transcript per million mapped reads (Florea et al. 2013). DEGs were identified using DESeq2 with the threshold of |log_2_(fold change)| ≥ 1 and FDR < 0.01 (Love et al. 2014).

For KEGG analysis (Kanehisa et al. 2008) (http://www.genome.jp/kegg/), we used KOBAS (Mao et al. 2005) software to test the statistical enrichment of DEGs in KEGG pathways.

### Analysis of metabolites in GTs

This part of work is done by the Allwegene Company in Beijing. GT samples were centrifuged at 13,800 × *g* for 15 min at 4 °C, and 50 mg of the precipitates was aliquoted into Eppendorf tubes followed by the addition of 700 *µ*L of extract buffer [3:1 (*v*/*v*) methanol/water, precooled to −40 °C containing an internal standard]. The samples were vortexed for 30 s and then homogenized at 35 Hz for 4 min and sonicated for 5 min in an ice-water bath. The homogenization and sonication steps were each repeated 3 times. The samples were incubated overnight at 4 °C on a shaker and centrifuged at 13,800 × *g* for 15 min at 4 °C. The supernatant was filtered through a 0.22 *µ*m microporous membrane. The filtrate was diluted by 10-fold with a 3:1 (*v*/*v*) methanol/water mixture (containing the internal standard). After vortex for 30 s, the mixture was transferred to 2 mL glass vials. An 80 *µ*L aliquot from each sample was collected and pooled to generate the quality control (QC) sample. The samples were stored at −80 °C.

The ultraperformance liquid chromatography–tandem mass spectrometer (UPLC–MS) analysis of metabolites was carried out on an EXIONLC System (Sciex Technologies, USA). Mobile Phase A consisted of 0.1% (*v*/*v*) formic acid in water, and Mobile Phase B consisted of acetonitrile. The column temperature was set at 40 °C. The autosampler temperature was set at 4 °C, and the injection volume was 2 *µ*L. A Sciex QTrap 6500+ (Sciex Technologies, USA) was used for assay development. Typical ion source parameters were as follows: ion spray voltage, +5,500/−4,500 V; curtain gas, 35 psi; temperature, 400 °C; ion source gas 1, 60 psi; ion source gas 2, 60 psi; and DP, ±100 V.

The Sciex Analyst Workstation (version 1.6.3) was used for multiple reaction monitoring (MRM) data acquisition and processing. MS raw data (.wiff) files were converted to text files using MSconventer. An in-house R program and database were used for peak detection and annotation.

### UHPLC analysis of flavonoids in GTs

To measure flavonoids, the ovary samples collected on the day of anthesis were ground into fine powder in liquid nitrogen using a pestle and mortar. One gram of sample was placed in a 50 mL brown centrifuge tube and mixed with 10 mL 50% (*v*/*v*) ethanol solution. The mixture was treated in an ultrasonic processor at a frequency of 55 Hz and a temperature of 50 °C for 50 min and then centrifuged at 6000 × *g* for 10 min. The supernatant was collected; ∼8 mL of each extract was filtered through a 0.22 *µ*m nitrocellulose filter and transferred to a 10 mL brown centrifuge tube, which was then placed in a rotary evaporator at 50 °C for vacuum concentration to 2 mL.

UHPLC analysis was performed in the Agilent 1290 Infinity II LC system. The mobile phase, consisting of Solvent A (acetonitrile) and Solvent B [0.1% (*v*/*v*) methanoic acid], was delivered at 1 mL/min. The binary gradient elution conditions were as follows: 5% A for 5 min, 18% A for 10 min, 22% A for 25 min, 18% A for 30 min, and 5% A for 40 min. Both the samples and authentic standards for isoquercitrin, delphinidin-3-*O*-glucoside, and astragalin were subjected to the same conditions.

### Y1H assay

The *CsTBH* CDS was cloned into a pJG vector with the *galactokinase 1* (*GAL1*) promoter, which served as the effector construct. The flavonoid biosynthesis gene (*4CL*, *FLS*, *F3H, F3′H*, and *BZ1*) promoter sequences were individually cloned into a pLacZi vector with the *LacZ* reporter gene. The vectors were transformed into competent cells of the yeast strain EGY48. The cells were selected on SD–Trp/−Ura medium, and positive colonies were spotted onto glucose plates (2% w/v) containing X-gal at 28 °C for 4 d to confirm the development of a blue color. The primers are listed in Supplemental Table S7.

### Electrophoretic mobility shift assay

EMSA was performed to validate the results from Y1H assay for *CsTBH*-targeted flavonoid biosynthesis genes (*4CL*, *FLS*, *F3H*, and *BZ1*). For each gene, a 29 bp sequence containing the binding *cis*-element in the promoter of the gene was labeled with biotin. MBP-TBH recombinant proteins were expressed in the *Escherichia coli* BL21 (DE3) strain and then purified using amylose magnetic beads (New England Biolabs, Cat # E8035S). DNA gel mobility shift assay was performed using the EMSA kit (Beyotime, China, Cat # GS009) following manufacturer's protocol. Briefly, the DNA probes and proteins were coincubated in the reaction buffer at room temperature. Specific competitor (nonbiotin) and nonspecific competitor (mutated) probes were added in the reaction mixture for competition reaction. After incubation, the reaction mixture was separated by 6% (*v*/*v*) native polyacrylamide gel, and then, labeled DNA was detected using the Amersham ImageQuant 800 system. The primers are listed in Supplemental Table S7.

### Dual-LUC transient expression assay

The promoter sequences (∼2,500 bp upstream from the transcription start) of flavonoid biosynthesis genes *4CL*, *FLS*, *F3H*, and *BZ1* were cloned into pGreenII 0800-LUC to generate reporter constructs. The CDS of *CsTBH* was integrated into pGreenII 62-SK to generate an effector construct. Coexpression studies were conducted in *N. benthamiana* L. leaves as previously described (Yotsui et al. 2013). Leaves transformed with reporters and an empty effector served as the negative controls. The activities of firefly LUC and Renilla luciferase (REN) were examined using the Dual-Luciferase Reporter Assay System (Promega, https://www.promega.com.cn/). The ratio of LUC/REN was calculated. The primers are listed in Supplemental Table S7.

### Statistical and sequence analyses

For pair-wise comparisons of means, *t* tests were performed. For test of means among 3 or more groups, 1-way ANOVA was performed.

### Accession numbers

The generated raw reads have been uploaded to the National Center for Biotechnology Information under accession number (PRJNA938582). Sequence data from this article can be found in the Cucurbit Genomics Database (http://cucurbitgenomics.org/). The accession numbers are listed in Supplemental Table S7.

## Supplementary Material

kiad236_Supplementary_Data

## Supplemental data

The following materials are available in the online version of this article.

**Supplemental Figure S1.** Heat map of expression levels of flavonoid biosynthetic pathway GT-enriched genes by RT-qPCR.

**Supplemental Figure S2.** EMSA showing CsTBH binding to the promoters of flavonoid-related genes.

**Supplemental Figure S3.** Analysis of VIGS efficiency in cucumber via *CsPDS*.

**Supplemental Table S1.** Summary of RNA-Seq read mapping results.

**Supplemental Table S2.** Summary of genes enriched in GTs.

**Supplemental Table S3.** Summary of metabolome profiling for GTs.

**Supplemental Table S4.** KEGG class analysis of genes enriched in GTs.

**Supplemental Table S5.** Summary of TFs enriched in GTs.

**Supplemental Table S6.** List of GTRGs.

**Supplemental Table S7.** Primers used in this study.
