# Supplementary material for: Novel players in organogenesis and flavonoid biosynthesis in cucumber glandular trichomes
Source: Plant Physiol. 2023 Apr 26;192(4):2723–36. doi: 10.1093/plphys/kiad236 (PMC10400037; doi:10.1093/plphys/kiad236)
Supplement: kiad236_Supplementary_Data [file kiad236_supplementary_data.zip › Supplemental Data.pdf]

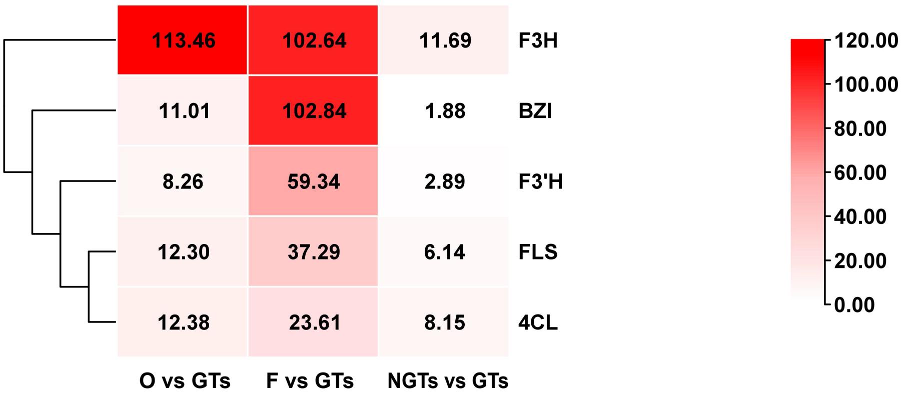

Supplemental Figure S1. Heatmap of expression levels of flavonoid biosynthetic pathway GTs-enriched genes by RT-qP-CR. The three columns represent gene relative expression of O vs GTs, F vs GTs and NGTs vs GTs, respectively. O = ovaries without trichomes; F = fruit flesh; GTs = glandular trichomes; NGTs = non-glandular trichomes.

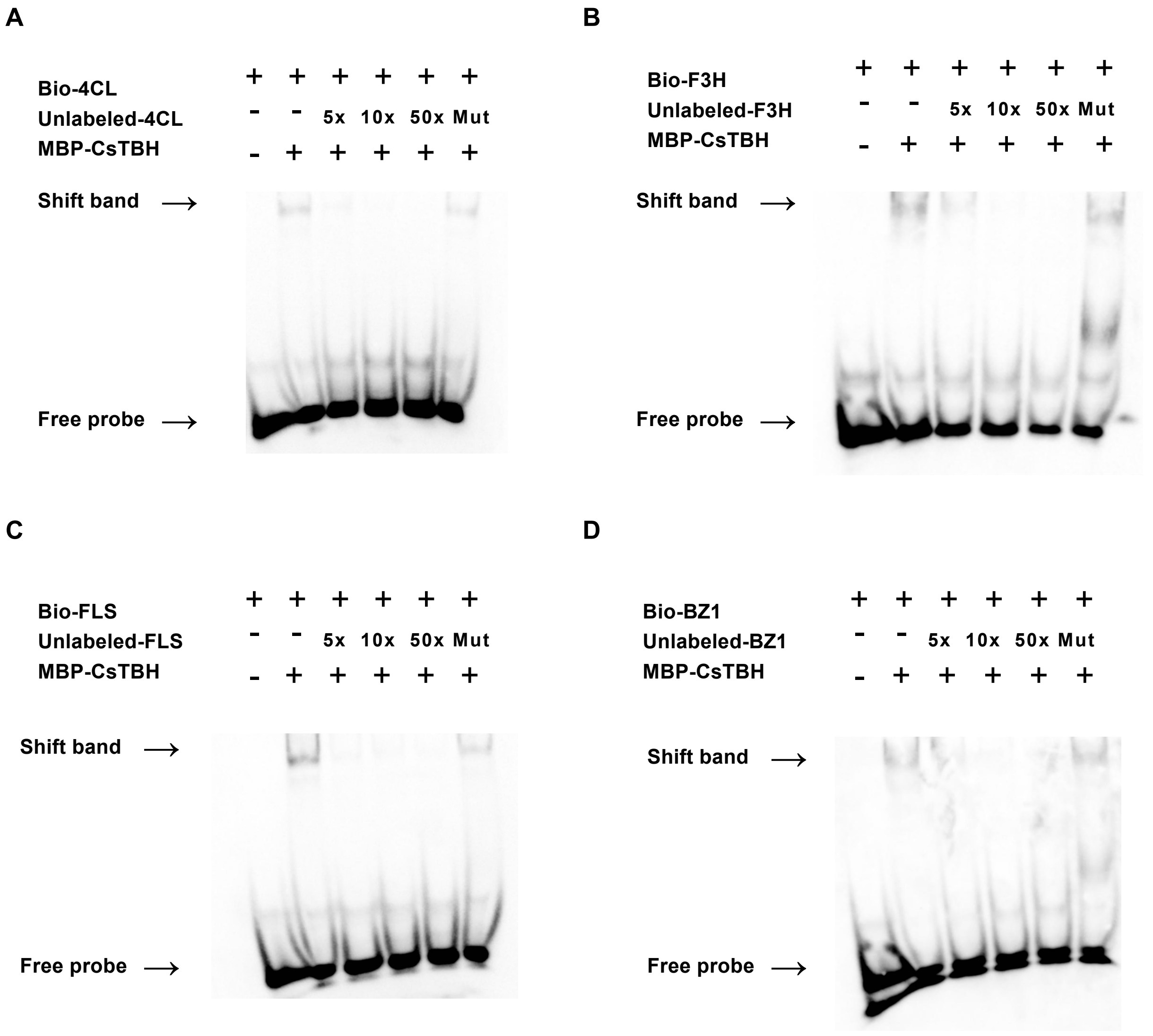

Supplemental Figure S2 EMSA showing CsTBH binding to the promoters of flavonoid-related genes. CsTBH binds to the promoters of *Cs4CL* (A), *CsF3H* (B), *CsFLS* (C), *CsBZ1* (D). Non-labeled probes (5x, 10x, 50x) were added as competitor.

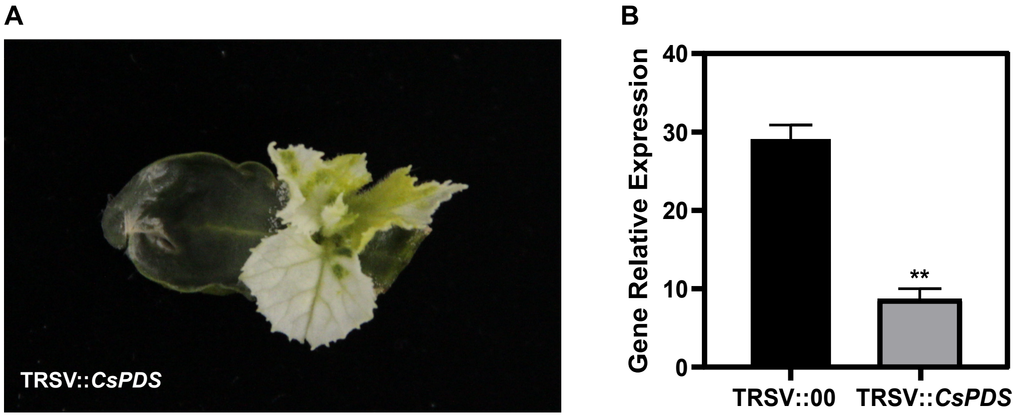

Supplemental Figure S3 Analysis of VIGS efficiency in cucumber via *CsPDS*. A. The phenotype of TRSV::*CsPDS* infected plant. B. Relative expression of *CsPDS* in TRSV::00 and TRSV::*CsPDS* infected plants. *CsTUA* served as the internal control. Three biological replicates were performed. Error bars represent SD from three biological repeats. ** indicates p-value < 0.01.
